# Enlarged Thyroid Due to Impacted Third Molar

**Published:** 1919-04

**Authors:** A. B. French


### ENLARGED THYROID DUE TO IMPACTED THIRD MOLAR.

BY A. B. FRENCH, D.D.S.

That local irritation may be the cause of an enlarged thyroid gland is indicated by the history of the following case:

Patient, male, age twenty-two, weight one hundred and thirty pounds, height five feet eight inches; presented September 14, 1917, for relief from pulpitis in upper right second bicuspid.

In making general survey of the mouth I noted the presence of all the third molars, except the lower right which was not in evidence.

The extensively enlarged (interfered with speech) gland being on the right side, led me to suspect a possible connection between the two.

Advised patient to call the attention of his physician to it, and see what he thought about it.

As might have been expected, the physician scoffed at the idea of any possible connection, giving the accepted causes, and so forth.

No improvement being noticeable under general treatment, and being encouraged by reference to the subject in an article by Dr. Cryer, I persuaded patient that inasmuch as it ought to be removed anyway and could not possibly do any harm, we take a chance in hope of benefiting the thyroid trouble.

Accordingly, on October 26, 1917, I removed the tooth.

Improvement in the patient's general condition was noticeable in ten days, and in six weeks there was a marked decrease in the size of the enlargement, treatment by physician having been discontinued from time of extraction.

At the present time, fourteen months after extraction, the enlargement can only be noticed by the closest observation and not be suspected except for the knowledge of its previous existence.

Patient is satisfied the tooth was the cause, which, of course, proves nothing.

All I myself am sure of is the correctness of the facts heretofore stated. To sum up: Where he had an impacted lower right third molar and an enlarged right thyroid gland, the latter disappeared following the removal of the former.—*Dental Digest.*

